# Human Cytomegalovirus Encodes a Novel FLT3 Receptor Ligand Necessary for Hematopoietic Cell Differentiation and Viral Reactivation

**DOI:** 10.1128/mBio.00682-18

**Published:** 2018-04-24

**Authors:** Lindsey B. Crawford, Jung Heon Kim, Donna Collins-McMillen, Byeong-Jae Lee, Igor Landais, Christine Held, Jay A. Nelson, Andrew D. Yurochko, Patrizia Caposio

**Affiliations:** a Vaccine and Gene Therapy Institute, Oregon Health & Science University, Portland, Oregon, USA; b Department of Microbiology & Immunology, Center for Molecular and Tumor Virology, Feist-Weiller Cancer Center, Louisiana State University Health Sciences Center, Shreveport, Louisiana, USA; Columbia University Medical College

**Keywords:** Flt3 receptor, human cytomegalovirus, hematopoiesis, pUL7, viral reactivation

## Abstract

The ability of human cytomegalovirus (HCMV) to reactivate from latent infection of hematopoietic progenitor cells (HPCs) is intimately linked to cellular differentiation. HCMV encodes UL7 that our group has shown is secreted from infected cells and induces angiogenesis. In this study, we show that UL7 is a ligand for Fms-like tyrosine kinase 3 receptor (Flt-3R), a well-known critical factor in HPC differentiation. We observed that UL7 directly binds Flt-3R and induces downstream signaling cascades, including phosphatidylinositol 3-kinase (PI3K)/Akt and mitogen-activated protein kinase (MAPK)/extracellular signal-regulated kinase (ERK) pathways. Importantly, we show that UL7 protein induces differentiation of both CD34^+^ HPCs and CD14^+^ monocytes. Last, we show that an HCMV mutant lacking UL7 fails to reactivate in CD34^+^ HPCs *in vitro* as well as in humanized mice. These observations define the first virally encoded differentiation factor with significant implications not only for HCMV reactivation but also for alteration of the hematopoietic compartment in transplant patients.

## IMPORTANCE

Human cytomegalovirus (HCMV) remains a significant cause of morbidity and mortality in allogeneic hematopoietic stem cell transplant recipients. CD34^+^ hematopoietic progenitor cells (HPCs) represent a critical reservoir of latent HCMV in the transplant population, thereby providing a source of virus for dissemination to visceral organs. HCMV reactivation has been linked to HPC/myeloid cellular differentiation; however, the mechanisms involved in these events are poorly understood at the molecular level. In this study, we show that a viral protein is a ligand for Fms-like tyrosine kinase 3 receptor (Flt-3R) and that the binding of HCMV UL7 to the Flt-3R triggers HPC and monocyte differentiation. Moreover, the loss of UL7 prevents viral reactivation in HPCs *in vitro* as well as in humanized mice. These observations define the first virally encoded differentiation factor with significant implications not only for HCMV reactivation but also for alteration of the hematopoietic compartment in transplant patients.

## INTRODUCTION

Human cytomegalovirus (HCMV) remains a significant cause of morbidity and mortality in allogeneic hematopoietic stem cell transplant (HSCT) recipients (1). In these patients, cytopenias occur as part of an “HCMV syndrome” defined by the presence of fever, viremia, and myelosuppression (2, 3). CD34^+^ hematopoietic progenitor cells (HPCs) provide a critical reservoir for HCMV, and infection of these cells may have both direct and indirect effects on hematopoiesis (4, 5; recently reviewed in reference 6). Several mechanisms may explain the deleterious effect of HCMV on bone marrow function, including altering hematopoiesis in infected cells and altering the cytokine expression program to affect the bone marrow microenvironment and differentiation of HPCs (7–10). Additionally, HCMV infection has also been associated with poor engraftment of HPCs (11, 12). Early studies using *in vitro* CD34^+^ HPC systems indicated that HCMV infection of CD34^+^ HPCs alters lymphoid and myeloid development (11, 13, 14). However, the mechanisms involved in these events remain unknown.

Several *in vitro* and *in vivo* models have shown that reactivation of latent virus requires stimulation of latently infected CD34^+^ HPCs by cytokines and growth factors that induce the myeloid differentiation events needed for production of infectious virus (15). Consistent with these observations, granulocyte colony-stimulating factor (G-CSF) mobilization of CD34^+^ HPCs in mice latently infected with HCMV induces an increase in myeloid cells in the peripheral blood, resulting in reactivation of virus in various tissue macrophages (16). The differentiation of CD34^+^ HPCs into fully differentiated tissue macrophages is a multistep process with each step requiring a specific and appropriate milieu of cytokines and cell-cell interactions. Similarly, the reactivation of latent HCMV is also a complex process integrally linked to the differentiation of the cells.

Over the past 2 decades, analysis of HCMV in CD34^+^ HPCs and myeloid lineage cells have identified several virally encoded genes associated with latency, including the UL133-138 locus (17–19), US28 (20–23), and LUNA (latency unique natural antigen) (24, 25). Expression of these viral genes has been detected during latent infection of CD34^+^ HPCs, and mutation of UL138, UL135, or US28 alters the ability of HCMV to establish latency in these cells. Moreover, these latency genes are hypothesized to promote and maintain the viral latent phenotype. Reactivation of HCMV from latency is a multistep process requiring the expression of viral genes in a cascade event in which immediate early (IE) genes are necessary to transcriptionally activate the early and late genes that encode proteins necessary for viral DNA replication and virion formation. Most of the characterization studies of the timing of viral gene expression during infection were performed in human fibroblasts (26). The temporal class of HCMV gene expression during reactivation in CD34^+^ HPCs remains unknown. The expression of IE genes is considered the primary event for reactivation; however, these genes can be expressed in cells that do not produce virus such as in monocytes where further myeloid differentiation events are necessary to activate the complete viral transcriptome.

UL7 is a member of the HCMV RL11 gene family and encodes a 222-amino-acid type I glycoprotein that is proteolytically cleaved to produce a heavily glycosylated ectodomain that is secreted into the supernatant of infected cells. The UL7 gene is kinetically expressed during the early-late phase of HCMV replication, it is nonessential for replication in human fibroblasts, and it is not expressed in CD34^+^ HPCs during latency (15, 26, 27). We have previously demonstrated that UL7 induces phosphorylation of signal transducer and activator of transcription factor 3 (STAT3) and extracellular signal-regulated kinase 1 and 2 (ERK1/2) mitogen-activated protein (MAP) kinases, resulting in the production of interleukin-6 (IL-6) in endothelial cells (27). Additionally, UL7 was observed to be a proangiogenic factor inducing angiogenesis in aortic endothelial cells (27).

In the current study, we examined the functional aspects of UL7 on HCMV reactivation in CD34^+^ HPCs as well as the effect of this protein on hematopoiesis. UL7 protein was found to bind Fms-like tyrosine kinase 3 receptor (Flt-3R), inducing differentiation of both CD34^+^ HPCs and blood monocytes, thus promoting myelopoiesis. We observed that deletion of UL7 in HCMV significantly reduced the ability of the virus to reactivate using both *in vitro* and *in vivo* latency models. These results indicate that UL7 is a Flt-3R ligand that is an important factor for HCMV reaction in CD34^+^ HPCs through the induction of myeloid cell differentiation.

## RESULTS

### UL7 is a ligand for Flt-3R and activates the PI3K/AKT and MAPK/ERK pathways.

**FIG 1:**
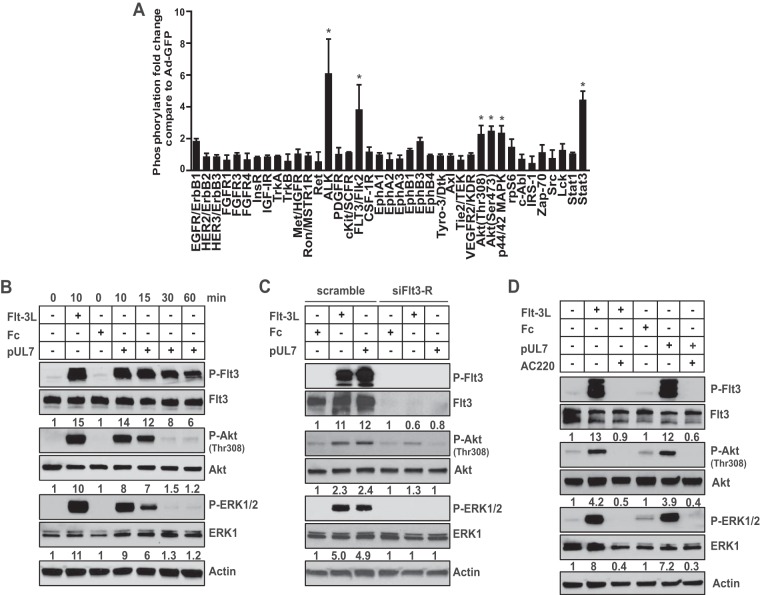
UL7 activates Flt-3R, inducing phosphorylation of Akt and ERK1/2. (A) Purified CD34^+^ HPCs were infected with adenovirus expressing UL7 (Ad-UL7) or GFP (Ad-GFP). Cell lysate were harvested at 24 h and analyzed using PathScan signaling antibody array. The experiment was performed with cells from two independent donors for each set of duplicate spots. Significant results (more than twofold induction) are marked with an asterisk; error bars show the standard errors of the means (SEM). (B) Protein lysates were generated and immunoblotted for phosphorylation of Flt-3R, Akt, and ERK1/2 from serum-starved RS4;11 cells stimulated (+) with 50 ng/ml of pUL7, Fc, or Flt-3L for the indicated time (in minutes). P-Flt3, phosphorylated Flt3; P-Akt, phosphorylated Akt; P-ERK1/2, phosphorylated ERK1/2. (C) RS4;11 cells were transiently transfected with Flt-3R siRNA or a scrambled siRNA for 24 h, and stimulated for 10 min. (D) RS4;11 cells were pretreated with AC220 (100 nM) for 1 h and then stimulated for 10 min. Protein lysates were generated and immunoblotted for phosphorylation of Akt and ERK1/2. Equal loading was confirmed by Flt-3R, Akt, ERK1, and actin antibody staining. Numbers under the immunoblots indicate relative phosphorylation of Flt-3R, Akt (Thr308), or ERK1/2 normalized to the amount of Flt3-R, Akt or ERK1 and to the value of untreated cells untreated or cells treated with Fc. Results are representative of three independent experiments.

Since HCMV UL7 induces signaling through the STAT3 and MAP kinase (MAPK)/ERK pathways, resulting in the production of IL-6 (27), which is important for both myeloid cell differentiation and viral reactivation, we initially examined the effect of UL7 expression in CD34^+^ HPCs on the activation of multiple receptor tyrosine kinases (RTKs) activated through these pathways. For these studies, CD34^+^ HPCs were transduced with an adenovirus vector expressing UL7 (Ad-UL7) followed by analysis of RTKs 24 h posttransduction using the PathScan RTK signaling antibody array that detects the phosphorylation of tyrosine residues in 28 RTKs and activation of 11 signaling nodes. Analysis of the results revealed a 5.8-fold increase in anaplastic lymphoma kinase (ALK) and a 3.4-fold increase in Flt-3R, as well as activation of intracellular Akt, ERK1/2, and STAT3 in comparison to a control adenovirus expressing green fluorescent protein (Ad-GFP) (Fig. 1A).

Flt-3R is predominantly expressed in hematopoietic lineage cells, and activation of this receptor mediates CD34^+^ HPC, lymphoid, and myeloid cell differentiation events (28–30). Additionally, phosphorylation of Flt-3R results in the activation of signaling in the Akt and ERK1/2 pathways. These observations suggested that UL7 may act as a Flt-3R ligand, activating the signaling events observed in CD34^+^ HPCs. In order to determine whether secreted UL7 binds Flt-3R to activate signaling, soluble Flt-3 ligand (Flt-3L), purified UL7 (pUL7), or a protein control (Fc) was added to cultures of bone marrow lymphoblast RS4;11 cells that express high Flt-3R levels (31), followed by analysis of Flt-3R signaling events. As shown in Fig. 1B, pUL7 induced phosphorylation of Flt-3R (14-fold compared to the Fc control), Akt (8-fold), and ERK1/2 (9-fold) within 10 min of stimulation. To determine whether the effects of UL7 were mediated through Flt-3R, RS4;11 cells were transfected with small interfering RNAs (siRNAs) targeting Flt-3R followed by administration of pUL7, Fc, or Flt-3L. As shown in Fig. 1C, Flt-3R siRNA significantly decreased the protein level of the receptor and that correlated with the reduction to basal levels of Akt or ERK1/2 phosphorylation induced by pUL7 or Flt-3L. The specificity of the Flt-3R siRNA is demonstrated in Fig. S1 in the supplemental material. To further confirm these results, RS4;11 cells were treated with the Flt-3R inhibitor quizartinib (AC220), followed by the addition of pUL7, Flt-3L, or Fc, and analysis of signaling was performed. As shown in Fig. 1D, addition of AC220 abolished the pUL7-induced phosphorylation of Flt-3R, Akt, and ERK1/2. Similar results were obtained in primary blood monocytes stimulated with Flt-3L, pUL7, or Fc in the presence or absence of AC220 (Fig. S2).

10.1128/mBio.00682-18.1 FIG S1  Specificity of Flt-3R siRNA. RS4;11 cells were transiently transfected with Flt-3R siRNA or scrambled siRNA, serum starved, and then stimulated with LPS (100 ng/ml) for 24 h. Protein lysates were generated and immunoblotted for phosphorylation of Akt and ERK1/2. The effect of the Flt-3R siRNA was confirmed by Flt-3R antibody staining. Equal loading was confirmed by Akt, ERK1, and actin antibody staining. Results are representative of three independent experiments. Download FIG S1, EPS file, 1.8 MB. Copyright © 2018 Crawford et al. 2018 Crawford et al. This content is distributed under the terms of the Creative Commons Attribution 4.0 International license.

10.1128/mBio.00682-18.2 FIG S2  UL7 activates ERK1/2 phosphorylation in primary monocytes. pUL7 (50 ng/ml) was used to treat fresh peripheral blood monocytes for 30 min. For a control for Flt-3R signaling, AC220 (10 nM) was used to pretreat cells for 1 h prior to the addition of pUL7 or Flt-3L. Protein lysates were generated and immunoblotted for phosphorylation of ERK1/2. Equal loading was confirmed by ERK1 and actin antibody staining. Results are representative of three independent experiments using samples from different donors. Download FIG S2, EPS file, 1.5 MB. Copyright © 2018 Crawford et al. 2018 Crawford et al. This content is distributed under the terms of the Creative Commons Attribution 4.0 International license.

**FIG 2:**
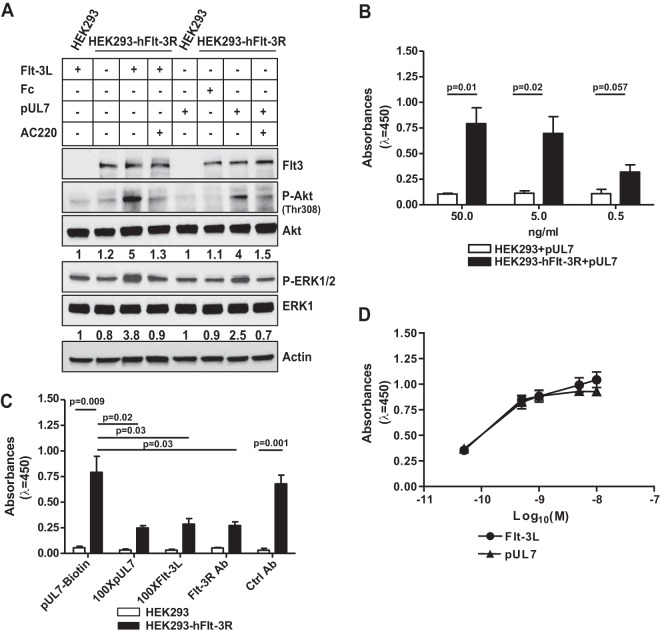
UL7 binds and signals through Flt-3R. (A) HEK-293 cells and HEK-293 cells expressing human Flt-3R (HEK-293-hFlt-3R) were serum starved, stimulated with 50 ng/ml of purified UL7 (pUL7), Fc, or Flt-3L for 10 min in the presence (+) or absence (−) of 100 nM AC220. Protein lysates were generated and immunoblotted for phosphorylation of Akt, and ERK1/2. Equal loading was confirmed by Flt-3R, Akt, ERK1, and actin antibody (Ab). Numbers under the blot indicate relative phosphorylation of Akt (Thr308) or ERK1/2 normalized to Akt or ERK1 amount and to the value of 293 cells treated with Flt-3L or Fc. Results are representative of three independent experiments. (B) HEK-293 and HEK-293-hFlt-3R cells were incubated with 50, 5, and 0.5 ng/ml of pUL7 or Fc for 1 h. The levels of pUL7 binding are expressed in absorbance units (λ = 450 nm). Values are means ± standard error of the means (SEM) (error bars) from three independent experiments. Statistical significance was determined using unpaired *t* test (*P* values are shown). (C) HEK-293 and HEK-293-hFlt-3R cells were incubated with biotinylated pUL7 (pUL7-biotin) in the presence of 100-fold excess of nonbiotinylated pUL7 (100XpUL7), or 100-fold excess Flt-3L (100XFlt-3L), or anti-Flt-3R antibody (anti-Flt-3R Ab), or a control antibody (Ctrl Ab). Values are means plus SEM (error bars) from three independent experiments. Statistical significance was determined using unpaired *t* test (*P* values are shown). (D) Serum-starved HEK-293-hFlt-3R cells were treated for 10 min with increasing concentrations of Flt-3L or pUL7 (M) (0.05 to 10 nM). Phosphorylation levels of Flt-3R were determined by PathScan phospho-Flt-3R (panTyr) ELISA. Values are means ± SEM (error bars) from three assays with each concentration measured in triplicate.

In order to determine whether pUL7-mediated ERK1/2 and AKT signaling is due to direct interaction with Flt-3R, stable HEK-293 cells expressing human Flt-3R (HEK-293-hFlt-3R) were made. As shown in Fig. 2A, pUL7 or Flt-3L, but not the Fc control, induced phosphorylation of Akt and ERK1/2 only in HEK-293-hFlt-3R cells, and this effect was blocked by treatment with AC220. HEK-293 cells do not express the receptor, and neither pUL7 nor Flt-3L was able to activate downstream signaling. In order to determine whether pUL7 directly binds to Flt-3R, biotinylated pUL7 was added to cultures of HEK-293-hFlt-3R or control cells and assessed for binding by an enzyme-linked immunosorbent assay (ELISA). As shown in Fig. 2B, pUL7 bound to the HEK-293-hFlt-3R cells, but not control cells, in a dose-dependent manner. In addition, a reduction in binding by 62% was observed in cells incubated with 100-fold excess of nonbiotinylated pUL7 and 63% with 100-fold excess of Flt-3L compared to HEK-293-hFlt-3R cells incubated with biotinylated pUL7 alone. Finally, treatment with the anti-Flt-3R antibody significantly (*P* = 0.03) decreased the binding of biotinylated pUL7 (Fig. 2C). In order to compare the relative activities of pUL7 and Flt-3L, HEK-293-hFlt-3R cells were stimulated with increasing concentrations (0.05 to 10 nM) of the two ligands, and Flt-3R phosphorylation was measured by a PathScan ELISA. As shown in Fig. 2D, the highest level of total tyrosine phosphorylation induced by pUL7 and Flt-3L was achieved at 10 nM after 10 min of stimulation, suggesting a similar mode of Flt-3R activation.

Together, these observations indicate that pUL7 is a ligand for Flt-3R able to activate the phosphatidylinositol 3-kinase (PI3K)/AKT and MAPK/ERK signaling pathways.

### UL7 induces myelopoiesis and promotes HCMV reactivation.

**FIG 3:**
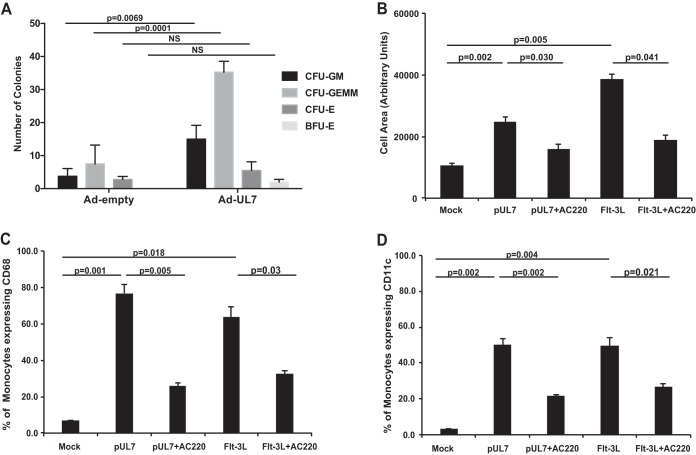
UL7 induces myelopoiesis and monocyte differentiation. (A) CD34^+^ HPCs were infected with adenovirus expressing UL7 (Ad-UL7) or empty adenovirus (Ad-Empty) for 24 h, sorted for viable, CD34^+^ HPCs, and plated in cytokine-rich methylcellulose. CFU for myeloid (granulocyte-macrophage [CFU-GM] and granulocyte-erythrocyte-monocyte-megakaryocyte progenitors [CFU-GEMM]) and erythroid (erythroid progenitors [CFU-E] and burst-forming units [BFU-E]) colonies were scored microscopically 14 days after infection. Statistical significance was determined using two-way analysis of variance followed by Sidak’s *posthoc* test (*P* values are shown; NS, not significant). (B) Peripheral blood monocytes were treated with pUL7 or Flt-3L (50 ng/ml) in the presence or absence of AC220 (100 nM), then plated on fibronectin-coated glass coverslips, and incubated for 6 days. Cells were fixed and visualized with a Leica TCS SP5 confocal microscope. The average size (in arbitrary units) of at least 50 monocytes for each experimental arm was determined from the captured images using ImageJ software. (C) Monocytes were incubated for 4 days with pUL7 or Flt-3L (50 ng/ml) in the presence or absence of AC220, then fixed, and stained with anti-CD68 Ab-conjugated to PE and analyzed by flow cytometry. (D) Monocytes were incubated for 4 days with pUL7 or Flt-3L (50 ng/ml) in the presence or absence of AC220, then fixed, and stained with anti-CD11c Ab conjugated to Alexa Fluor 700 and analyzed by flow cytometry. In each graph, values are means ± SEM (error bars) from three independent experiments using different human donors. Statistical significance was determined using one-way analysis of variance followed by Tukey’s posthoc test (*P* values are shown).

Since Flt-3R plays a critical role in hematopoiesis, we examined the ability of CD34^+^ HPCs to form hematopoietic colonies in methylcellulose cultures in the presence of UL7. We compared cells transduced with an adenovirus without an insert (Ad-Empty) to the Ad-UL7. As shown in Fig. 3A, expression of UL7 substantially increased the ability of CD34^+^ HPCs to form myeloid colonies (*P* = 0.0001 for CFU-GEMM [CFU for granulocyte-erythrocyte-monocyte-megakaryocyte progenitors] and *P* = 0.0069 for CFU-GM [CFU for granulocytes-macrophages]).

In order to determine whether UL7 also induces monocyte differentiation, pUL7 was added to cultures of fresh peripheral blood monocytes, followed by analysis of cellular differentiation. As shown in Fig. 3B, treatment with pUL7 and the control Flt-3L induced monocyte-to-macrophage differentiation as measured by increasing cell size over time. Treatment with AC220 abrogated the differentiation process. These results were confirmed by the expression of the macrophage differentiation marker CD68 (Fig. 3C) and the macrophage/dendritic cell maturation marker CD11c (Fig. 3D) following pUL7 or Flt-3L treatment with or without AC220 treatment.

**FIG 4:**
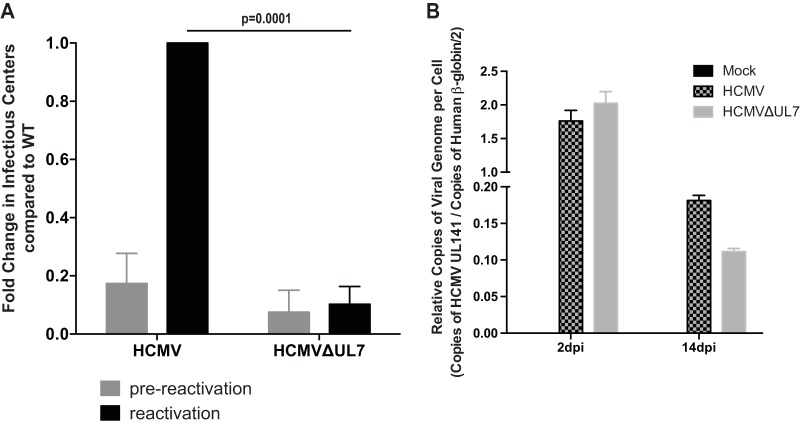
UL7 is required for HCMV reactivation *in vitro*. (A) Pure populations of CD34^+^ HPCs infected with WT HCMV or ΔUL7 mutant were isolated by FACS at 42 hpi and maintained in LTBMC medium. At 14 days postinfection (dpi), viable CD34^+^ HPCs were seeded onto fibroblast monolayers plated in 96-well dishes by limiting dilution in cytokine-rich medium for reactivation. An equivalent number of cells was mechanically disrupted and seeded in parallel to determine the infectious virus present in the cultures prior to reactivation (prereactivation). The frequency of infectious center formation pre- and postreactivation was determined 14 days later from the number of GFP-positive (GFP^+^) wells at each dilution using extreme limiting dilution analysis. The values shown are the fold change in reactivation normalized to that of WT HCMV postreactivation. Values are means plus SEM (error bars) from three independent experiments using different human donors. Statistical significance was determined using unpaired *t* test (*P* values are shown). (B) Total DNA from primary CD34^+^ HPC cultures was extracted at 2 dpi and 14 dpi (12 days after latency culture initiation, latency established) and HCMV genomes were quantified using quantitative PCR with primers and probe specific for the *UL141* gene. Viral genomes synthesized during infection in CD34^+^ HPCs were normalized to the total cell number determined using human β-globin as a reference. Data shown are the mean values for three replicate quantitative PCR (qPCR), and error bars represent standard deviations. Data shown are representative of three independent experiments using different donor CD34^+^ HPCs. See also Fig. S3 in the supplemental material.

Since HCMV reactivation from latency is integrally linked to differentiation, we examined the effect of UL7 mutation on the ability of virus to reactivate in latently infected CD34^+^ HPCs. In these studies, CD34^+^ HPCs were infected with an HCMV that expresses GFP with a deletion in UL7 (HCMVΔUL7) or wild-type (WT) HCMV expressing GFP. HCMV-infected CD34^+^ HPCs were purified by fluorescence-activated cell sorting (FACS) at 42 h postinfection (hpi) and seeded into long-term bone marrow culture (LTBMC) over stromal cell support (32). After 12 days of culture in LTBMC, HPCs latently infected with HCMV were seeded onto monolayers of permissive fibroblasts in cytokine-rich media to promote myeloid differentiation. As shown in Fig. 4A and Fig. S3, WT HCMV but not HCMVΔUL7 reactivated in these cultures. Analysis of viral DNA in CD34^+^ HPCs infected with both WT HCMV and HCMVΔUL7 at 2 through 14 days postinfection (dpi) indicated similar amounts of HCMV genomes in both cell populations (Fig. 4B and Fig. S3). These results indicate that the inability of HCMVΔUL7 to reactivate in CD34^+^ HPCs is not due to the loss of genome in latently infected cells.

10.1128/mBio.00682-18.3 FIG S3  UL7 is required for reactivation, but not genome maintenance. CD34^+^ HPCs were infected with HCMV or HCMV lacking UL7 for 42 h, sorted for pure CD34^+^ GFP^+^ HPCs and plated for long-term culture on stromal cell support. (A, C, and E) After 12 days (14 dpi), reactivation was assessed by coculture on fibroblasts from three independent experiments. (B and D) DNA from a subset of cells was prepared using the two-step TRIZOL method, and viral genomes were analyzed by qPCR. Download FIG S3, EPS file, 1.4 MB. Copyright © 2018 Crawford et al. 2018 Crawford et al. This content is distributed under the terms of the Creative Commons Attribution 4.0 International license.

**FIG 5:**
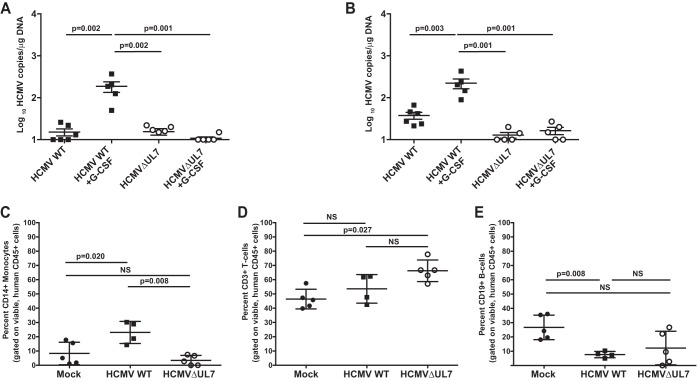
UL7 is required for HCMV reactivation and myelopoiesis *in vivo*. Sublethally irradiated NOD-*scid* IL2Rγcnull mice were engrafted with CD34^+^ HPCs (huNSG) and subsequently injected with human fibroblasts previously infected with WT HCMV or ΔUL7 mutant as indicated. huNSG mice injected with uninfected fibroblasts served as a negative control (*n* = 5). At 8 weeks postinfection, viral reactivation was triggered by treating latently infected WT HCMV and HCMVΔUL7 (*n* = 5) animals with G-CSF and AMD-3100. At 1 week posttreatment, mice were euthanized and tissues were harvested. Total genomic DNA was isolated from spleen (A) and liver (B) tissues, and HCMV genomes were quantified using quantitative PCR with primers and probe specific for the *UL141* gene. Statistical significance was determined using two-way analysis of variance, followed by Bonferroni’s *posthoc* test (*P* values are shown). Peripheral blood was harvested from mock-infected huNSG mice (*n* = 5), huNSG mice latently infected with WT HCMV (*n* = 4), or HCMVΔUL7-infected huNSG mice (*n* = 5) at 8 weeks postinfection and analyzed by flow cytometry for the relative percentage of human CD14^+^ monocytes (C), CD3^+^ T cells (D), and CD19^+^ B cells (E). All samples were treated with red blood cell (RBC) lysis buffer, blocked with human and mouse serum, and stained with antibodies specific for human CD3, CD14, CD19, and CD45, and mouse CD45. Data shown for each individual huNSG mouse were gated on total, viable, mouse CD45^−^, human CD45^+^ leukocytes. Error bars represent standard errors of the means. Statistical significance was determined using one-way analysis of variance, followed by Tukey’s posthoc test (*P* values are shown; NS, not significant).

To extend the results of Fig. 4, we examined the ability of HCMVΔUL7 to reactivate in a humanized mouse model. In this model, CD34^+^ HPCs are engrafted into NOD-*scid*IL2Rγc null mice (huNSG), followed by HCMV infection (16). After viral latency is established, HCMV can be reactivated and disseminated into tissues through infected macrophages following treatment of mice with G-CSF and AMD3100 (16). As shown in Fig. 5, both WT HCMV and HCMVΔUL7 were able to establish a low level of latent infection in the spleen (Fig. 5A) and liver (Fig. 5B). However, only WT HCMV, not HCMVΔUL7, reactivated in these tissues. The lack of reactivation in mice infected with HCMVΔUL7 was not due to differences in the levels of human cell engraftment compared to the animals infected with WT HCMV, as shown in Table 1. Interestingly, analysis of human hematopoietic cell populations in WT HCMV-infected huNSG mice compared to HCMVΔUL7-infected huNSG mice indicated that CD14^+^ monocytes/macrophages (Fig. 5C), but not lymphocyte populations (CD3^+^ T cells and CD19^+^ B cells) (Fig. 5D and E), were increased in the peripheral blood in WT HCMV-infected mice. HCMVΔUL7-infected mice exhibited levels of CD14^+^ cells similar to those of uninfected mice (Fig. 5C). The inability of HCMVΔUL7 to increase the CD14^+^ monocyte population in tissues compared to WT HCMV correlates with the ability of pUL7 to induce myelopoiesis *in vitro*. These results indicate that HCMV UL7 induces the differentiation of CD34^+^ HPCs into myeloid cells that are critical for reactivation of latent virus in tissues.

**TABLE 1:** Human cell engraftment in huNSG mice prior to infection

huNSG cohort	Mock-infected mice (*n* = 5)		WT HCMV-infected mice (*n* = 5)		HCMVΔUL7-infected mice (*n* = 5)	
	% positive	SD (%)	% positive	SD (%)	% positive	SD (%)
huCD45	40.2	26.7	30.3	16.1	37.2	13.1
huCD3	45.4	12.2	46.7	25.7	50.6	3.9
huCD19	47.0	11.0	46.8	22.9	41.8	5.4
huCD14	1.3	1.0	0.9	0.6	1.8	0.8

## DISCUSSION

In summary, our results indicate that UL7 signals through the Flt-3R to activate the PI3K/AKT and Ras/Raf/MAPK pathways in both CD34^+^ HPCs and blood monocytes, resulting in differentiation of these cells. pUL7 was sufficient to induce CD34^+^ HPC myelopoiesis and monocyte differentiation, and deletion of the gene in the virus altered CD14^+^ hematopoietic populations in tissues. These observations, in combination with the inability of HCMVΔUL7 to reactivate in latently infected CD34^+^ HPCs, suggest that UL7 mediates the cellular differentiation necessary for viral reactivation from latency. We propose a model in which inflammatory cytokines such as IL-6 and tumor necrosis factor alpha (TNF-α) trigger reactivation of latent HCMV in CD34^+^ HPCs by activating immediate early (IE), early, and late genes. The late gene UL7 is then secreted from cells and through an autocrine manner binds to Flt-3R on HCMV-infected cells, inducing myeloid cell differentiation into macrophages, thus allowing full expression of the viral transcriptome and production of progeny virus.

UL7 belongs to the RL11 family (RL11-13, UL1, UL4-11, RL6, RL5A) that encodes a characteristic domain, called RL11D, which has limited sequence homology to immunoglobulin domains and to the immunomodulatory E3 proteins of adenoviruses (33). All members of the family are dispensable for virus growth *in vitro*, and most of the viral genes are predicted to encode membrane glycoproteins (34). Functional data for these genes are limited. RL13 has been described as a potent inhibitor of viral replication (35) and an immunomodulatory protein (36). UL1 is a viral tropism factor (37), UL8 was reported to decrease the release of proinflammatory factors (38), and UL10 (39) and UL11 (40) were observed to modulate T cell activation. Among RL11D genes, UL7 exhibits high sequence conservation across different CMV genotypes (97 to 100% intragenotype conservation and 83 to 93% intergenotype conservation), suggesting a critical role for UL7 in viral fitness (41).

Reactivation of HCMV *in vitro* can be triggered from early myeloid cells (CD34^+^ and CD14^+^ cells) by differentiation (42). Early studies demonstrated that treatment of monocytes with a mixture of cytokines that promote macrophage differentiation could trigger the reactivation of IE gene expression (43) and, in some instances, infectious particles (44). More recently, studies have now established that HCMV reactivation occurs in myeloid progenitors differentiated into dendritic cells (DCs) by specific cytokine stimulation, including IL-6 (45, 46). We previously reported that UL7 induces secretion of IL-6 in endothelial cells (27). Interestingly, treatment of long-term cultures of nonadherent, experimentally latent monocytes in media supplemented with IL-6 has been shown to induce HCMV reactivation (47), and similarly, the lipopolysaccharide (LPS)-mediated reactivation of HCMV from experimentally latent immature DCs has been shown to be dependent on IL-6 (46). An interesting aspect of these studies was the phenotype of the differentiated monocyte: the cells reactivating HCMV coexpressed both macrophage (CD68) and DC (CD83) markers. In accordance with these data, we observed an increase of CD68 in monocytes and CD11c DCs after 4 days of stimulation with pUL7 (Fig. 3C and D).

Among herpesviruses, HCMV UL7 is not the only viral factor able to induce cellular differentiation. Kaposi’s sarcoma-associated herpesvirus (KSHV) encodes a viral IL-6 that shares 25% homology with human IL-6 and is sufficient to drive blood to lymphatic endothelial cell differentiation through the JAK2/STAT3 and PI3K/Akt signaling pathways (48). KSHV also encodes a viral microRNA (miRNA), miR-K12-11, that shares 100% seed sequence homology with hsa-mir-155, an oncogenic human miRNA that functions as a key regulator of hematopoiesis and B-cell differentiation (49). An *in vivo* study validated miR-K12-11 as a functional ortholog of miR-155 in the context of hematopoiesis by demonstrating that miR-K12-11 induces B-cell expansion and potentially KSHV-associated lymphomagenesis (49). Our new data now demonstrate that UL7 represents the first HCMV-encoded protein to regulate hematopoietic differentiation events, in particular by favoring myeloid cell differentiation.

Flt-3R is a member of the type III RTK family and is known to play a crucial role during hematopoiesis (50). In normal bone marrow, Flt-3R is selectively expressed on CD34^+^ hematopoietic progenitor cells and immature hematopoietic progenitors, including B-lymphoid progenitors, myeloid precursors, and monocytes (51, 52). Furthermore, it has been experimentally shown that CD34^+^ bone marrow cells can give rise to two different populations according to the level of Flt-3R expression: cells expressing high levels of the receptor mature into granulocyte-macrophage colonies in *in vitro* myeloid colony assays, while cells that express low levels of the receptor preferentially mature into burst-forming unit erythroid colonies (53). Flt-3R is also expressed in other hematopoietic organs, such as the spleen, liver, thymus, lymph nodes, and placenta, and is found in blood-forming organs, such as gonads and brain (30). Here, we found that UL7 is able to bind Flt-3R on cells expressing the Flt-3R (Fig. 2B) and is sufficient to induce both myeloid colony formation from HPCs (Fig. 3A) and monocyte-to-macrophage differentiation (Fig. 3B to D).

A commonly encountered clinical manifestation of HCMV infection in hematopoietic stem cell transplant (HSCT) and solid-organ transplant recipients is myelosuppression (1, 54). Despite the well-described clinical relevance of hematopoietic cell lineage infection by HCMV, the fundamental mechanisms underlying myelosuppression and graft failure are poorly understood. Several mechanisms may explain the deleterious effect of HCMV on bone marrow function, including altering hematopoiesis in infected cells and an altered cytokine expression program that affects the bone marrow microenvironment. In regard to direct effects, our new data suggest that expression of UL7 induces myeloid colony formation (Fig. 3A) and is essential *in vivo* for the HCMV-driven maturation and expansion of CD14^+^ monocytes (Fig. 5C). We hypothesize that secretion of HCMV UL7 directly promotes myelopoiesis, which may explain the increase in CD14^+^ cells in tissues of humanized mice infected with WT HCMV, but not HCMVΔUL7. Our observation that UL7 regulates hematopoiesis has implications on the potential role of HCMV in hematological diseases.

In conclusion, HCMV reactivation is a multistep process that involves changes in the cellular environment. An incorrect assumption would be that there is one de facto mechanism required for viral reactivation. The induction of IE gene expression is predicted to be essential for initiating HCMV reactivation; however, expression of these genes does not dictate that infectious virus will be produced from latently infected cells. Our data indicate that other viral gene products, such as UL7, and cellular interactions that lead to myeloid cell differentiation are important for driving HCMV reactivation and production of progeny virus. Future studies will elucidate the role of UL7 in HCMV reactivation and development of hematopoietic disease.

## MATERIALS AND METHODS

### Cells.

CD34^+^ hematopoietic progenitor cells (HPCs) were isolated from fetal liver obtained from Advances Bioscience Resources and cultured as previously described (18). Peripheral blood mononuclear cells (PBMCs) were obtained from healthy donors as approved by the Institutional Review Board for the Protection of Human Research Subjects at Louisiana State University Health Sciences Center—Shreveport, and follows all guidelines outlined by the Health Insurance Portability and Accountability Act. PBMCs were isolated using a Ficoll Histopaque 1077 gradient (Sigma-Aldrich) and then washed with irrigation-grade saline, and the monocytes were purified over a Percoll gradient (Amersham Pharmacia). Purified monocytes were washed in RPMI 1640 medium and suspended in RPMI 1640 medium (Cellgro) supplemented with 1% human AB serum (Sigma-Aldrich). RS4;11 cells (bone marrow lymphoblasts) and MRC-5 cells (fetal human lung fibroblasts) were obtained from the American Type Culture Collection. RS4;11 cells were grown in RPMI 1640 medium (HyClone) medium supplemented with 10% fetal bovine serum (FBS) (HyClone), 4.5 g/liter glucose, l-glutamine, and sodium pyruvate, and antibiotics (penicillin [10 units/ml]-streptomycin [10 µg/ml]). MRC-5 cells were cultured with Dulbecco’s modified Eagle’s medium (DMEM) (Cellgro) supplemented with 10% FBS, penicillin, streptomycin, and l-glutamine. Human embryonic kidney 293 cell line (HEK-293) and HEK-293/Cre4 cell line (Microbix Biosystems Inc.) were cultured in minimum essential medium (MEM) (Cellgro) supplemented with 10% FBS, penicillin, streptomycin, and l-glutamine. The M2-10B4 murine stromal cell line expressing human interleukin-3 (IL-3) and granulocyte colony-stimulating factor (G-CSF) and the S1/S1 murine stromal cell line expressing human IL-3 and stem cell factor (SCF) were obtained from Stem Cell Technologies.

RS4;11 cells were transfected using the Nucleofector kit R (Lonza). After addition of a negative-control small interfering RNA (siRNA) (100 pmol) (Silencer Select [catalog no. 4390843; ThermoFisher Scientific]) or a mix of two FLT3 siRNAs (50 pmol each) (Silencer Select [catalog no. s5291 and s5292; ThermoFisher]), the mixture was transferred to a nucleofection cuvette and electroporated using a Nucleofector 2 device (Lonza) and program T-016.

Stable transfected HEK-293 cells expressing human Flt-3 receptor (Flt-3R) (HEK-293-hFlt-3R) were generated by transfection of FLT3 (green fluorescent protein [GFP]-tagged)-human fms-tyrosine kinase 3 plasmid (catalog no. RG211459; OriGene Technologies) with Lipofectamine 2000 (Life Technologies) followed by G418 selection (800 µg/ml).

### Viruses.

The HCMV TB40/E bacterial artificial chromosome (BAC) was previously engineered to express green fluorescent protein (GFP) as a visual marker of infection (55). Recombinant HCMV TB40/EΔUL7 was constructed by E/T recombination using pOri-FRT vector (56) as a template to generate the PCR product and the primers UL7-FRTkan forward (5′-GGCACTGTTTGAGCATGACTGTTTCCAAACCGTAACGTGGTAAATAATCAGCTCGGTACCCGGGGATCTT-3′) and UL7-FRTkan reverse (5′-GATGTAGTCATTGTTGGTACAAAACCTTCTCCCTGATAAAAAACACATTAACGCAGCTTCAAAAGCGCTCT-3′). After selection of recombinant BACs on the basis of kanamycin resistance, the Kan^r^ marker was removed by flp-mediated recombination. BAC integrity was examined by enzyme digest analysis and sequencing of the entire viral genome by next-generation sequencing. Virus was reconstituted by transfecting the BAC genomes into MRC-5 fibroblasts. Plaque purification was performed. and HCMV TB40/EΔUL7 stocks and titers were generated as previously described (27).

### Adenovirus production.

Recombinant adenoviruses were produced by pAdTet7 UL7HA or GFP or empty vector cotransfection of 293-Cre cells with adenovirus (Ad) DNA (Ad5-ψ5), as previously described (27).

### PathScan arrays.

The PathScan RTK signaling antibody array kit (Cell Signaling Technology) is a slide-based antibody array product founded upon the sandwich immunoassay principle. Briefly, 1 × 10e6 CD34^+^ HPCs were infected at a multiplicity of infection (MOI) of 1,000 with adenovirus vector expressing GFP (Ad-GFP) or UL7 (Ad-UL7), sorted after 24 h for viable CD34^+^ HPCs, washed in phosphate-buffered saline (PBS), lysed in 1× cell lysis buffer (Cell Signaling Technology), and processed according to the manufacturer’s protocol. The PathScan phospho-Flt3 (panTyr) is a solid-phase sandwich enzyme-linked immunosorbent assay (ELISA) (Cell Signaling Technology). Briefly, 8 × 10e5 cells were plated in 24-well flat-bottom plate and grown overnight at 37°C with 5% CO_2_. The cells were starved in 0.5% FBS for 24 h before treatment with Flt-3 ligand (Flt-3L) or purified UL7 (pUL7) (0.05 to 10 nM) for 10 min at 37°C with 5% CO_2_. The cells were washed in PBS, lysed in cell lysis buffer (Cell Signaling Technology), and quantified by Pierce BCA protein assay kit (ThermoFisher). Lysates (20 µg) were processed according to the manufacturer’s protocol.

### Reagents.

Recombinant UL7 protein from TB40E (TB40E, accession number EF999921; UL7 protein, accession number ABV71537.1) was generated at RayBiotech. Briefly, UL7 TB40E gene (nucleotides 1 to 411) was synthesized and cloned into pExpR7 (with a C-terminal mouse IgG Fc tag and a His tag for protein affinity purification). The pExpR7 was transfected into HEK-293 cells, and the UL7 recombinant protein was recovered from the cell culture medium and purified using a Ni column. The purified protein was subjected to 12% SDS-PAGE analysis, Coomassie blue staining, and Western blotting using UL7 antibody (27). Biotinylated UL7 was generated using DSB-X biotin protein labeling kit (ThermoFisher Scientific) according to the manufacturer’s protocol. Quizartinib (AC220) was purchased at Selleckchem and used at 100 nM based on previous cytotoxicity studies in RS4;11 and THP-1 cells (31); recombinant mouse IgG Fc and recombinant human Flt-3L were purchased at R&S Systems.

### Immunoblotting.

Extracts were run on 8 to 12% sodium dodecyl sulfate (SDS)-polyacrylamide gels, transferred to Immobilon-P transfer membranes (Millipore Corp.), and visualized with antibodies specific for phospho-ERK1/2 (Thr202/Tyr204; Cell Signaling Technology), ERK1 (C16; Santa Cruz), phospho-Akt (Thr308; Cell Signaling Technology), Akt (C73H10; Cell Signaling Technology), phospho-Flt3 (Tyr591; Cell Signaling Technology), FLT3 (8F2; Cell Signaling Technology), and actin (C4; Millipore).

### Cell binding assay.

Confluent HEK-293 and HEK-293-hFlt-3R cells in 96-well trays were washed twice with MEM containing 1% (wt/vol) bovine serum albumin (BSA) and incubated for 1 h with 0.5, 5, or 50 ng/ml of biotinylated pUL7 in the same MEM diluent. Horseradish peroxidase (HRP)-conjugated streptavidin (ThermoFisher) diluted 1:1,000 was added to the cells for 45 min, and then the cells were washed twice in MEM and reacted with 100 µl of 3,3′,5,5′-tetramethylbenzidine (TMB) substrate (ThermoFisher Scientific) for 30 min. The reaction was stopped with 100 µl of 2 M sulfuric acid, and the absorbance was determined at a wavelength (λ) of 450 nm. For competition assay, confluent HEK-293 and HEK-293-hFlt-3R cells in 96-well trays were preincubated with 100-fold excess of nonbiotinylated pUL7 (5 µg/ml), Flt-3L (5 µg/ml), anti-Flt-3R (8F2; Cell Signaling Technology) (1:100), or control antibody (1:100) for 1 h, then washed with MEM, and incubated with 50 ng/ml of biotinylated pUL7.

### Limiting dilution reactivation assay.

CD34^+^ HPCs were infected at an MOI of 3 for 42 h. Following infection, pure populations of viable, infected (GFP-positive) CD34^+^ HPCs were isolated by fluorescence-activated cell sorting (FACS) using a FACSAria II system (Becton Dickinson) and propidium iodine (PI) for viability and an allophycocyanin (APC)- or phycoerythrin (PE)-Cy7-conjugated antibody specific to CD34 (clone 561; BioLegend). Long-term cultures were maintained in Myelocult supplemented with hydrocortisone in Transwells above an irradiated M2-10B4 and S1/S1 stromal cell monolayer for 12 days (32). After 12 days in long-term bone marrow culture, infected hematopoietic cell populations were serially diluted twofold in reactivation medium, namely, RPMI 1640 medium supplemented with 20% FBS, 1 mM sodium pyruvate, 2 mM l-glutamine, 100 U/ml penicillin, 100 µg/ml streptomycin, 15 ng/ml granulocyte colony-stimulating factor (G-CSF), 15 ng/ml granulocyte-macrophage colony-stimulating factor (GM-CSF), and 15 ng/ml Flt-3L (all cytokines purchased from R&D Systems, Minneapolis, MN). Fibroblasts were examined microscopically for GFP expression for up to 40 days. To differentiate virus made as a result of reactivation from virus preexisting in the cell cultures, an equal number of cells was serially diluted and plated on fibroblasts after being mechanically disrupted. The frequency of infectious center production during the culture period was measured using a limiting dilution assay as previously described (55).

### CD34^+^ HPC and monocyte differentiation assays.

Purified CD34^+^ HPCs were infected with Ad-UL7 or Ad-Empty for 24 h at an MOI of 1,000, and then 5 × 10e2 cells/ml were seeded in methylcellulose medium, MethoCult H4434 (Stem Cell Technologies) in a minimum of three replicate dishes per group. Hematopoietic colonies were scored microscopically 7 and 14 days later. Fresh peripheral blood monocytes were treated with pUL7 (50 ng/ml) or Flt-3L (50 ng/ml) in the presence or absence of AC220 (100 nM), plated on fibronectin-coated glass coverslips, and incubated for 6 days. Cells were then fixed with 4% paraformaldehyde (Fisher Scientific) and visualized with a Leica TCS SP5 confocal microscope (Leica Microsystems). The average size (in arbitrary units) of at least 50 monocytes for each experimental arm was determined from the captured images using ImageJ software.

### Flow cytometry analysis.

Monocytes were incubated for 4 days with pUL7 (50 ng/ml) or Flt-3L (50 ng/ml) in the presence or absence of AC220 (100 nM) and then fixed with 4% paraformaldehyde (Fisher Scientific). Cells were treated with an Fc receptor (FcR) blocking reagent (MACS), followed by staining with anti-CD68 antibody conjugated to PE (BioLegend) or anti-CD11c antibody conjugated to Alexa Fluor 700 (BioLegend). After antibody (Ab) staining, the cells were analyzed by flow cytometry using a FACSCalibur system (BD Biosciences). Human cell reconstitution and specific human cell populations were determined by flow cytometry on freshly isolated mononuclear cells obtained from blood from humanized or control mice. Samples were treated with red blood cell lysis buffer, washed with PBS, and stained with the viability dye Zombie Aqua (BioLegend) prior to antibody staining. Nonspecific antibody binding was blocked prior to and during staining using human and mouse serum. Flow cytometry analysis was performed using antibodies specific for the human cell surface markers CD3 (clone UCHT1), CD14 (M5E2 or HCD14), CD19 (HIB19), CD45 (HI30), and the murine cell surface marker CD45 (all from BioLegend). All samples were fixed with 2% neutral buffered formalin prior to analysis. All data were collected and analyzed using an LSRII flow cytometer equipped with FACS Diva (Becton Dickinson) and FlowJo v10 (TreeStar).

### Engraftment and infection of humanized mice.

All animal studies were carried out in strict accordance with the recommendations of the American Association for Accreditation of Laboratory Animal Care (AAALAC). The protocol was approved by the Institutional Animal Care and Use Committee (protocol 0922) at Oregon Health and Science University. NOD-*scid* IL2Rγ_c_^null^ mice were maintained at a pathogen-free facility at Oregon Health and Science University in accordance with procedures approved by the Institutional Animal Care and Use Committee. Both sexes of animals were used. Humanized mice were generated as previously described (16, 18). The animals (12 to 14 weeks postengraftment) were treated with 1 ml of 4% thioglycolate (Brewer’s medium; BD) by intraperitoneal (i.p.) injection to recruit monocyte/macrophages. At 24 h posttreatment, mice were infected with wild-type HCMV TB40E or HCMVΔUL7-infected fibroblasts (approximately 10^5^ PFU of cell-associated virus per mouse) via i.p. injection. A control group of engrafted mice were mock infected using uninfected fibroblasts. The virus was reactivated as previously described (16, 18).

### Quantitative PCR for viral genomes.

Total DNA from mouse tissues was extracted using the DNAzol kit (Life Technologies), and processed as previously described (16, 18). Total DNA from primary CD34^+^ HPC cultures was extracted with the ZR-Duet DNA/RNA miniprep kit (Zymo) according to the manufacturer’s directions. Primers and a probe recognizing HCMV UL141 were used to quantify HCMV genomes (probe, CGAGGGAGAGCAAGTT; forward primer, 5′-GATGTGGGCCGAGAATTATGA; reverse primer, 5′-ATGGGCCAGGAGTGTGTCA). Viral genomes in humanized mice were normalized to 1 µg input DNA. Viral genomes synthesized during infection in CD34^+^ HPCs were normalized to the total cell number determined using human β-globin as a reference (probe, GGACAGATCCCCAAAGGACT; forward primer, 5′-TTAGGGTTGCCCATAACAGC; reverse primer, 5′-TTGGACCCAGAGGTTCTTTG). The reaction was initiated using TaqMan Fast Advanced master mix (Applied Biosystems) activated at 95°C for 10 min, followed by 40 cycles (1 cycle consists of 15 s at 95°C and 1 min at 60°C) using a StepOnePlus TaqMan PCR machine. Results were analyzed using ABI StepOne software.

### Statistical analysis.

Data are expressed as the means ± standard errors of the means (SEM) or mean ± standard deviations (SD). Statistical analysis was performed using GraphPad Prism (v6) for comparison between groups using an unpaired Student’s *t* test, one-way analysis of variance (ANOVA) with Tukey’s posthoc test or two-way ANOVA with Bonferroni’s or Sidak’s *posthoc* test as indicated. A *P* value of <0.05 or lower was considered significant as indicated.

### Data availability.

UL7 protein from HCMV TB40E can be downloaded from GenBank (GenBank accession number ABV71537.1).
